# Metabolomic analysis to predict the onset and severity of necrotizing enterocolitis

**DOI:** 10.1186/s12876-024-03453-y

**Published:** 2024-10-26

**Authors:** Laura Moschino, Giovanna Verlato, Matteo Stocchero, Giuseppe Giordano, Paola Pirillo, Marta Meneghelli, Silvia Guiducci, Miriam Duci, Francesco Fascetti Leon, Eugenio Baraldi

**Affiliations:** 1 University of Padova, Department of Women’s and Children’s Health, Padova, Italy; 2 University Hospital of Padova, Neonatal Intensive Care Unit, Padova, Italy; 3 Institute of Pediatric Research, Città della Speranza, Padova, Italy; 4 Laboratory of Mass Spectrometry and Metabolomics, Institute of Pediatric Research, Padova University Hospital, Padova, Italy; 5 Pediatric Surgery, Padova University Hospital, Padua, Italy

**Keywords:** Necrotizing enterocolitis, Preterm neonate, Biomarker, Mass-spectrometry, Metabolome

## Abstract

**Background:**

Necrotizing enterocolitis (NEC) is the most devastating gastrointestinal (GI) emergency in preterm neonates. Untargeted metabolomics may allow the identification of biomarkers involved in NEC pathophysiology.

**Methods:**

We conducted a prospective study including preterm infants born at < 34 gestational weeks (GWs) whose urine was longitudinally collected at birth (< 48 h, T0) and at 14 (T1) and 28 days (T2). Neonates were followed for their development of NEC, spontaneous intestinal perforation (SIP), or other GI conditions and compared to those of matched healthy controls. Urine samples were investigated by untargeted metabolomic analysis based on mass-spectrometry.

**Results:**

Thirty-five patients with NEC, 5 patients with SIP, 14 patients with other GI diseases and 113 controls were enrolled and selected for metabolomic analysis on the basis of their clinical characteristics and available samples. Considering urine samples at T0, the one-class classification approach was able to correctly classify 16/20 subjects (80%) who developed NEC, 3/3 (100%) who developed SIP and 5/7 subjects (71.4%) with other GI pathologies as not belonging to the control group. Neonates with surgical NEC had higher N-acetylaspartic acid, butyrylcarnitine and propionylcarnitine levels than did those with medical NEC. Considering the time evolution of the urinary metabolome, the NEC and control groups showed differences independently of the time point.

**Conclusions:**

The urinary metabolome is closely associated with the underlying GI disease from birth. Urinary metabolic features characterize NEC patients from healthy controls until 28 days of life. The early urinary metabolome has the potential to predict surgical NEC. Future studies are needed to validate our results.

**Supplementary Information:**

The online version contains supplementary material available at 10.1186/s12876-024-03453-y.

## Background

Necrotizing enterocolitis (NEC) is a devastating gastrointestinal emergency of preterm neonates, affecting approximately 6% of very low birth weight infants (VLBW, < 1500 g) and 7% (2–13%) of extremely low gestational age neonates (ELGANs) [1, 2]. The mortality rate can reach 50% in surgical patients with a BW < 1000 g and 80% in those with a fulminant course. Additionally, NEC is associated with increased morbidity in survivors, including intestinal failure due to short bowel syndrome, cholestasis, failure to thrive, and neurodevelopmental sequalae [3, 4].

NEC can manifest within various stages along a continuum of bowel disease. The famous classification proposed in 1978 by Bell and colleagues, with the subsequent modification [5] by Walsh and Kliegman in 1986 [6], is still the most widely used by clinicians worldwide. With this staging system based on pure clinical and radiological signs, however, it is often difficult to differentiate NEC from other gastrointestinal (GI) diseases, particularly spontaneous intestinal perforation (SIP) [7–9], self-resolving feeding intolerance, and paralytic ileus due to sepsis [10].

As clinical evaluation alone may be unreliable, neonatologists usually support their suspicion of NEC with several laboratory tests, despite the majority of alterations remaining aspecific and having low sensitivity [11–14].

Metabolomics, the youngest of the four major “omics” sciences, enables us to depict the ultimate phenotypic expression of the ongoing biochemical response to a stimulus [15]. Indeed, as the last downstream products of gene transcription and enzymatic pathways, metabolites provide the closest picture of an organism’s phenotype after interaction with the environment [16, 17]. This technique can be applied to different biological fluids, such as those noninvasively collected from preterm neonates (umbilical cord blood, plasma, urine, stool, tracheal aspirate, etc.), as the amount required for the analysis is very small (20 μL-300 μL) [17, 18]. Additionally, by the *untargeted approach,* the entire set of metabolites present in a biological sample can be extensively and comprehensively analysed without any a priori hypotheses [16]. Thus, new potential *profiles* of biomarkers can be discovered, revealing unknown pathogenetic mechanisms of a disease [16].

Despite the continuous evolution of metabolomic studies in neonatal research, few studies have investigated the application of these methods to diagnosing NEC and, especially, to its differential diagnostic conditions, such as SIP [19].

A recent systematic review conducted by our group [20] highlighted the presence of only five studies applying untargeted metabolomics (43 cases, 95 preterm controls) for NEC prediction or diagnosis (Bell’s stage ≥ II). The studies were cross-sectional, with prospective collection of samples and retrospective analysis using either nuclear magnetic resonance (NMR) spectroscopy or ultra-performance liquid chromatography‒mass spectrometry (UPLC-MS) applied to urinary or fecal samples. In these studies, metabolites belonging to pathways related to the inflammatory response and intestinal permeability (linoleate metabolism, C21-steroid hormone biosynthesis, leukotriene metabolism, the formation of prostaglandins from arachidonate, sphingomyelins, and ceramides) [21, 22], as well as to energy depletion (amino acids) [23–25], were hypothesized to be characteristic of affected infants. Interestingly, no studies have evaluated the metabolic differences between preterm infants with NEC and those with SIP or other gastrointestinal conditions or have focused on biomarkers of NEC severity.

An updated systematic review on targeted and untargeted metabolomics in NEC also highlighted the main metabolomic alterations involved in amino acid composition (in blood and urine), fatty acid metabolism (in blood), and less homogeneous findings in feces [26].

Given the scarcity of studies on this topic, we investigated the metabolic profile of infants who developed NEC to identify early predictive biomarkers of the disease and its severity. The primary aim of this study was to apply untargeted metabolomics based on mass spectrometry (MS) to early urine samples to identify potential biomarkers of NEC development in respect to a healthy state and to SIP or other GI conditions. The secondary aims were to explore the evolution of the urinary metabolome during the first month of life in neonates who developed NEC vs healthy controls and to characterize potential prognostic metabolic profiles of NEC severity (i.e., surgical NEC vs medical NEC).

## Materials and methods

### Study design and population

We conducted a single-centre study at the Neonatal Intensive Care Unit (NICU) of Padova University Hospital (Veneto, Italy) from January 2020 to July 2022. All infants who were admitted to the NICU and who were born at < 34^+0^ gestational weeks (GW) or who had congenital heart disease (CHD) were enrolled. The exclusion criteria were major congenital anomalies or chromosomal abnormalities, isolated structural abnormalities of the gut (i.e., omphalos or gastroschisis), or refusal of consent. Patients were prospectively followed for their possible development of NEC, SIP, or other GI conditions until discharge, transfer to another unit or hospital, or death. Infants not developing these conditions were considered healthy controls.

This was a pilot study applying an untargeted metabolomic approach, for which there was no a priori hypothesis of the number of variables characterizing each study group. Therefore, the sample size was based on previous studies on the topic [20, 23, 24, 27] and on the incidence of NEC in our center (approximately 10 cases/year, thus making 20 NEC patients suitable for explorative data analysis).

For each patient, clinical and demographic characteristics, as well as laboratory data, were recorded on a preformed electronic case report form (eCRF) on the REDCap platform. Plasma, urine and faecal samples were noninvasively collected at birth (within 48 h, T0), at 14 (T1) and 28 DOL (T2), at 2 months (T2 months) and at 36 weeks of corrected gestational age (cGA) (T36). In the suspicion of NEC, SIP or other GI diseases with a presentation similar to that of NEC, additional plasma, urine and faecal samples were collected at symptom onset and then weekly until resolution. More details on the data and sample collection can be found in the Supplemental Material.

NEC was defined according to the modified Bell’s stage criteria by Walsh and Kliegman [6]. In our Unit, medical NEC is usually defined as NEC Bell’s stage IA to IIB, while patients undergoing surgery are classified as NEC Bell’s stage IIIA and IIIB, as described by Neu and Walker in 2011 [28]. In particular, in the case of suspected onset of signs and symptoms of NEC (abdominal distension, bilious aspirates, bloody stools with or without systemic signs of instability), patients are managed according to our local protocol (algorithm in [29]).

Spontaneous isolated intestinal perforation (SIP) was defined by sudden clinical deterioration, associated with the presence of free intraperitoneal air (pneumoperitoneum) on abdominal X-ray without evidence of pneumatosis or signs of NEC in the case of laparotomy.

Functional paralytic ileus due to sepsis was defined as intolerance to oral intake (increase in gastric residuals > 50% of the volume of the preceding feed, with abdominal distension, and consequent interruption of enteral feeding) with ongoing late-onset sepsis (Vermont Oxford Network VON Criteria) [30]) and without any sign of mechanical intestinal obstruction.

Minimal enteral feeding (MEF) was defined as trophic feeds with a total volume of ≤ 24 ml/kg/day, while full enteral feeding (FEF) was defined as feeds with an enteral volume of 150 ml/kg/day.

Other clinical variables, such as prenatal flow alterations, preterm premature rupture of membranes (PPROM), intrauterine growth restriction (IUGR), and hemodynamically significant patent ductus arteriosus (HSPDA), were defined according to local protocols.

**Fig. 1:**
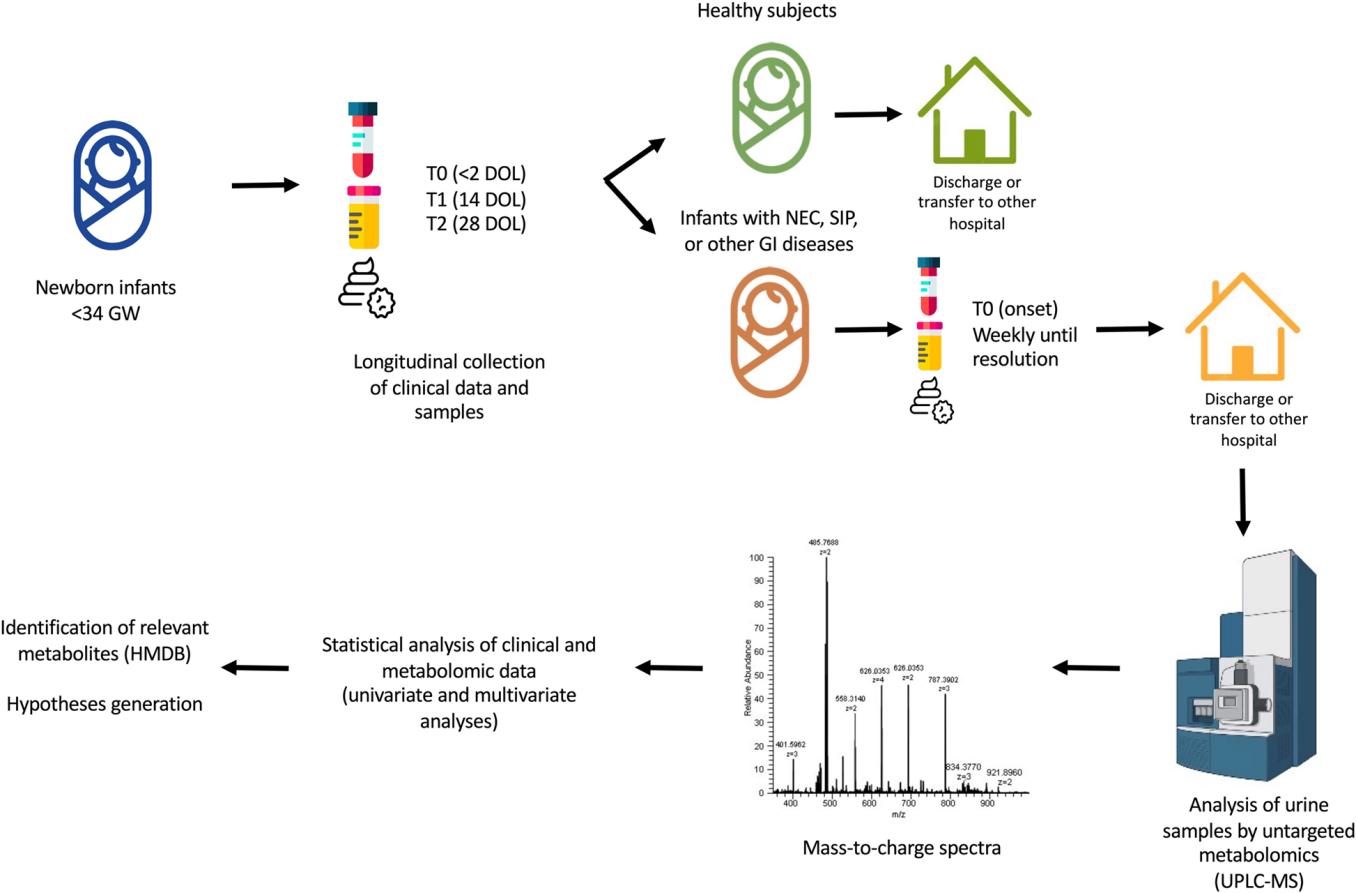
Diagram flow of the study design:from each enrolled patient, samples of plasma, urine and stools were collected at the timepoints T0, T14 and T28; patients were then followed for their possible development of NEC, SIP or other GI conditions until discharge or transfer to other hospital; urine samples were analysed by untargeted metabolomics using UPLC-MS; statistical data analysis of clinical and metabolomic data derived from mass-to-charge spectra identified potential relevant metabolites of NEC development and severity. Images from Freepik and BioRender.com

Figure 1 shows a diagram of the study design.

### Metabolomic and statistical analyses

Urine samples were analysed at the Mass Spectrometry and Metabolomics Laboratory of the Women’s and Children’s Health Department (Paediatric Research Institute, IRP, University of Padova, Italy). Untargeted metabolic profiling was performed in positive and negative electrospray ionization (ESI + , ESI-) mode on an Acquity Ultra Performance Liquid Chromatography (UPLC) system (Waters, U.K.) coupled to a quadrupole time-of-flight (QToF) Synapt XS HDMS mass spectrometer (Waters MS Technologies, Ltd., Manchester, U.K.). After the raw data were extracted, quality controls and blanks were used to calibrate and filter out the data. More details about the metabolomics investigation can be found in the Supplemental Material.

Categorical data were investigated by Fisher’s exact test, whereas t tests or Mann‒Whitney tests were applied to continuous normally or nonnormally distributed data, respectively. The normality of the data distribution was assessed by the Shapiro‒Wilk test.

Since the urinary metabolome is strongly dependent on the characteristics of the subjects, a suitable procedure of matching was applied to avoid bias due to differences in the perinatal and neonatal characteristics of the groups under investigation [31].

Different approaches were applied to investigate the urinary metabolomics data.

Specifically, one-class classification (OCC), including both univariate and multivariate methods in the framework of model population analysis (MPA) [32], was applied to discriminate controls from the other subjects. OCC can be successfully applied in clinical applications to solve problems where a reference group is compared with observations that do not belong to well-defined classes, or with a number which is unsuitable to allow the application of discriminant techniques. In this case, OCC provided the number of observations predicted as control or as not belonging to the control group. In the case of two-group comparisons, PLS for classification (PLSC) with stability selection [33] and the Mann‒Whitney test controlling the false discovery rate by the Benjamini‒Hochberg method [34] were used. PLS for designed experiments (PLS-doe)[35] and linear mixed-effects modelling (LME) for longitudinal data [36] were applied to study the time evolution at the three time points. More details about the statistical data analysis can be found in the Supplemental Material.

Data analysis was performed by R-functions developed in-house using the R 4.0.4 platform (R Foundation for Statistical Computing).

Clinical and metabolomic data have been deposited in the Mendeley Data Repository (Moschino, L; Stocchero, M (2024), “METNEC Study”, Mendeley Data, V1, 10.17632/83myp4xfcn.1).

#### Ethics approval and consent to participate

Informed consent to participate was obtained from the parents or legal guardians of any participant under the age of 16.

The protocol of this study was written according to the principles of Good Clinical Practice of the European Union and Helsinki Declaration and was approved by the Institutional Review Board of Padova University Hospital (5128/AO/21, 4374/AO/17).

No funding sources supported this study.

## Results

### Clinical characteristics of the patients and healthy controls

During the study period, 35 patients with NEC, 5 patients with SIP, 14 patients with other GI diseases involved in the differential diagnosis of NEC (i.e., septic paralytic ileus, isolated rectal bleeding), and 113 healthy controls were recruited. The main clinical and laboratory characteristics of the unmatched initial groups are reported in Table 1. These findings show that both the NEC and SIP groups have a high burden of morbidity, with a prolonged time to reach full enteral feeding and a high risk of post-disease strictures (up to 18.5% and 20%, respectively, in our cohort).

**Table 1:** Main perinatal and neonatal characteristics of the different groups (NEC patients, SIP patients, patients with other GI conditions, controls). The data are expressed as the absolute number (%) or as the median (IQR)

	**NEC Group** (*n* = 35)	**SIP Group** (*n* = 5)	**Other Gastrointestinal conditions** (*n* = 14)	**Control Group** (*n* = 113)
**Type of disease**	**Bell’s stage of NEC** IA 6 (17.1%) IB 2 (5.7%) IIA 12 (34.3%) IIB 1 (2.9%) IIIA 6 (17.1%) IIIB 8 (22.9%)	NA (1 case with development of NEC IIIB after SIP)	**Final diagnosis** Septic paralytic ileus 2 (14.3%) Isolated rectal bleeding 8 (57.1%) Others (volvulus, obstruction) 4 (28.6%)	NA
**Sex**				
Male	23 (65.7%)	4 (80%)	5 (35.7%)	63 (55.8%)
Female	12 (34.3%)	1 (20%)	9 (64.3%)	50 (44.2%)
**GA at birth** (weeks)	26.6 (25–30.1)	26.4 (26.3–28.1)	30.3 (27.6–32.6)	30 (28.1–32)
**Birth weight** (grams)	855 (752.5–1230)	790 (740–825)	1353 (816–1610)	1230 (950–1550)
**IUGR**	9 (25.7%)	2 (40%)	2 (14.3%)	24 (21.2)
**Apgar at 5 min**	7 (6–8)	7 (6–7)	8 (7–8)	8 (7–8)
**Ethnicity**				
Caucasic	33 (94.3%)	4 (80%)	11 (78.6%)	96 (85%)
African	1 (2.9%)	1 (20%)	2 (14.3%)	11 (9.7%)
Asiatic	1 (2.9%)	0 (0%)	1 (7.1%)	6 5.3%)
**Mode of delivery**				
Caesarean section	29 (82.9%)	4 (80%)	12 (85.7%)	100 (88.5%)
Vaginal delivery	6 (17.1%)	1 (20%)	2 (14.3%)	13 (11.5%)
**Prenatal steroids**				
Complete	20 (57.2%)	5 (100%)	10 (71.4%)	85 (75.2%)
Incomplete	6 (17.1%)	0 (0%)	1 (7.1%)	16 (14.2%)
None	9 (25.7%)	0 (0%)	3 (21.5%)	12 (10.6%)
**Treatment with surfactant**	24 (68.6%)	5 (100%)	9 (64.3%)	61 (54%)
**HsPDA**	18 (51.4%)	2 (40%)	4 (28.6%)	29 (25.7%)
**EOS**				
No	26 (74.2%)	5 (100%)	10 (71.4%)	90 (79.6%)
Suspected	8 (22.9%)	0 (0%)	4 (38.6%)	21 (18.6%)
Definitive	1 (2.9%)	0 (0%)	0 (0%)	2 (1.8%)
**Total days of ATBs for EOS**	7 (3–8)	3 (3–5)	6 (4–7)	4 (3–6)
**Postnatal systemic steroids**	6 (17.1%)	1 (20%)	1 (7.1%)	14 (12.4%)
**Human milk at start of feeding**	25 (71.4%)	4 (80%)	9 (64.3%)	68 (60.2%)
**Total days to reach FEF**	37.5 (26.7–65.5)	47 (41–114)	29 (19–48)	12 (8–21)

*Abbreviations*: *ATBs* antibiotics, *EOS* early onset sepsis, *FEF* full enteral feeding, *GA* gestational age, *HsPDA* haemodynamically significant patent ductus arteriosus, *IUGR* intrauterine growth restriction

Among the 35 NEC patients, 27 had Bell’s stage ≥ II NEC (11 who were medically treated and 16 who were surgically treated). Due to the small size of the SIP group, a statistical comparison between the NEC Bell’s stage ≥ II patients and the SIP patients was not performed. The main clinical and laboratory characteristics of these two groups are reported in Table 2. All these patients were treated with fasting and triple antibiotic therapy (usually vancomycin or ampicillin, ceftazidime or gentamicin and metronidazole).

**Table 2:** The main clinical and laboratory characteristics of neonates affected by Bell’s stage ≥ II NEC and by SIP within 48 h before and after onset, clinical management and potential complications. The data are expressed as the absolute number (%) or as the median (IQR)

	**NEC ≥ II** (*n* = 27)	**SIP** (*n* = 5)
**Type of disease**	**Bell’s stage of NEC** IIA 12 (37.5%) IIB 1 (3.1%) IIIA 6 (18.8%) IIIB 8 (25%)	
**DOL at disease onset**	10 (7–23.5)	4 (4–5)
**Fulminant onset (less than 48 h between onset and surgery/death)**	10 (37%)	0 (0%)
**Feeding with only human milk at onset**	8 (29.6%)	3 (60%)
**Total enteral amount at onset (ml/kg/day)**	140 (89.2–160)	28.5 (14.2–42.7)
**Transfusion of RBC within 48 h before onset**	7 (25.9%)	2 (40%)
**Days of NPO for the disease**^a^	16 (11–21)	15 (7–10)
**Days of ATBs for the disease**	15 (11.5–18.5)	13 (10–14)
**Haematocrit** (%)	31.9 (27.3–35)	34.1 (33–36.1)
**Lowest WBC** (/mmc)	6510 (4140–12620)	6820 (6250–6970)
**Lowest neutrophil count** (/mmc)	2970 (1690–7740)	5680 (4715–32725)
**Lowest platelet count** (/mmc)	153000 (58000–307000)	66000 (34000–172000)
**Highest CRP** (mg/L)	40 (8.6–80)	46.9 (8.8–52)
**Lowest albumin** (g/L)	27.5 (22–32.5)	23 (22–27)
**CRP/Albumin ratio**	1 (0–3.75)	2 (0–2)
**Surgery for NEC/SIP**	16 (59.3%)	5 (100%)
**Type of surgery for NEC/SIP** n (% of those with surgery)		
PD	2^b^ (12.5%)	2 (40%)
ExLap	9 (56.3%)	1 (20%)
PD followed by ExLap	5 (31.2%)	2 (40%)
**Intestinal involvement at ExLap or autopsy** n (% of those explored)		
Ileal	9 (60%)	1 (20%)
Colic	1 (6.7%)	2 (40%)
Pan-NEC	5 (33.3)	
**Haemoculture positive**	5 (18.5%) *1 St. aureus, 1 Klebsiella pneumonia ESBL, 1 Ent. Cloacae, 1 Str. Haemolyticus, 1 E. coli*	1 (20%) *Candida albicans*
**Peritoneal fluid culture positive**	7 (25.9%) *1 Klebsiella pneumoniae, 1 Cl. Perfrigens and St. aureus, 3 Ent. Faecalis, 1 St. hominis, 1 Cl. Perfrigens*	1 (20%) *Candida parapsilosis*
**Stenosis post-NEC/SIP**	5 (18.5%)	1 (20%)
**Death**	6 (22.2%)	0 (0%)

*Abbreviations*: *ATBs* antibiotics, *CRP* C-reactive protein, *ExLap* exploratory laparotomy, *NEC* necrotizing enterocolitis, *NPO* niil per os, *PD* peritoneal drainage, *SIP* spontaneous intestinal perforation, *WBC* white blood cells

^a^Including days of NPO for subsequent surgeries or complications of NEC

^b^One death before performing ExLap, autopsy not performed

### Data analysis of the urinary metabolome

Since the urinary metabolome is strongly dependent on the perinatal and neonatal characteristics of the recruited subjects, a suitable and strict matching procedure was applied to groups of subjects with similar characteristics to avoid bias in the data analysis. Additionally, only neonates with enough urine for the metabolomic analysis were considered. Specifically, the NEC group comprised 20 cases, and the control group comprised 17 neonates after matching for the main perinatal and neonatal characteristics (GA, BW, SEX, PROM, IUGR, EOS, HSPDA, delivery, Apgar 5’, prenatal steroids, surfactant, outborn/inborn) (Table S1 of the Supplemental material), with a significance level of 0.05. The NEC group included 7 neonates with Bell’s stage I NEC, 6 neonates with Bell’s stage II NEC and 7 neonates with Bell’s stage III NEC. Additionally, 3 subjects with SIP and 7 subjects with other GI diseases whose urine samples were collected at T0 were considered for the first step of the analysis. These latter groups were found to have similar GA and BW to those of the control group. After data preprocessing, two datasets, one composed of 1002 variables from the data acquired in negative ionization mode (*NEG dataset*) and one composed of 1086 variables from the data acquired in positive ionization mode (*POS dataset*), were obtained.

#### Urinary metabolome at birth (T0)

As a preliminary data analysis, the one-class classification (OCC) was applied to compare healthy controls with the other groups. The models were able to correctly predict that 16 out of 20 (80%) subjects with NEC, 5 out of 7 (71.4%) subjects with other GI pathologies and 100% of the subjects who developed SIP did not belong to the control group (Figure S1 of the Supplemental material).

**Fig. 2:**
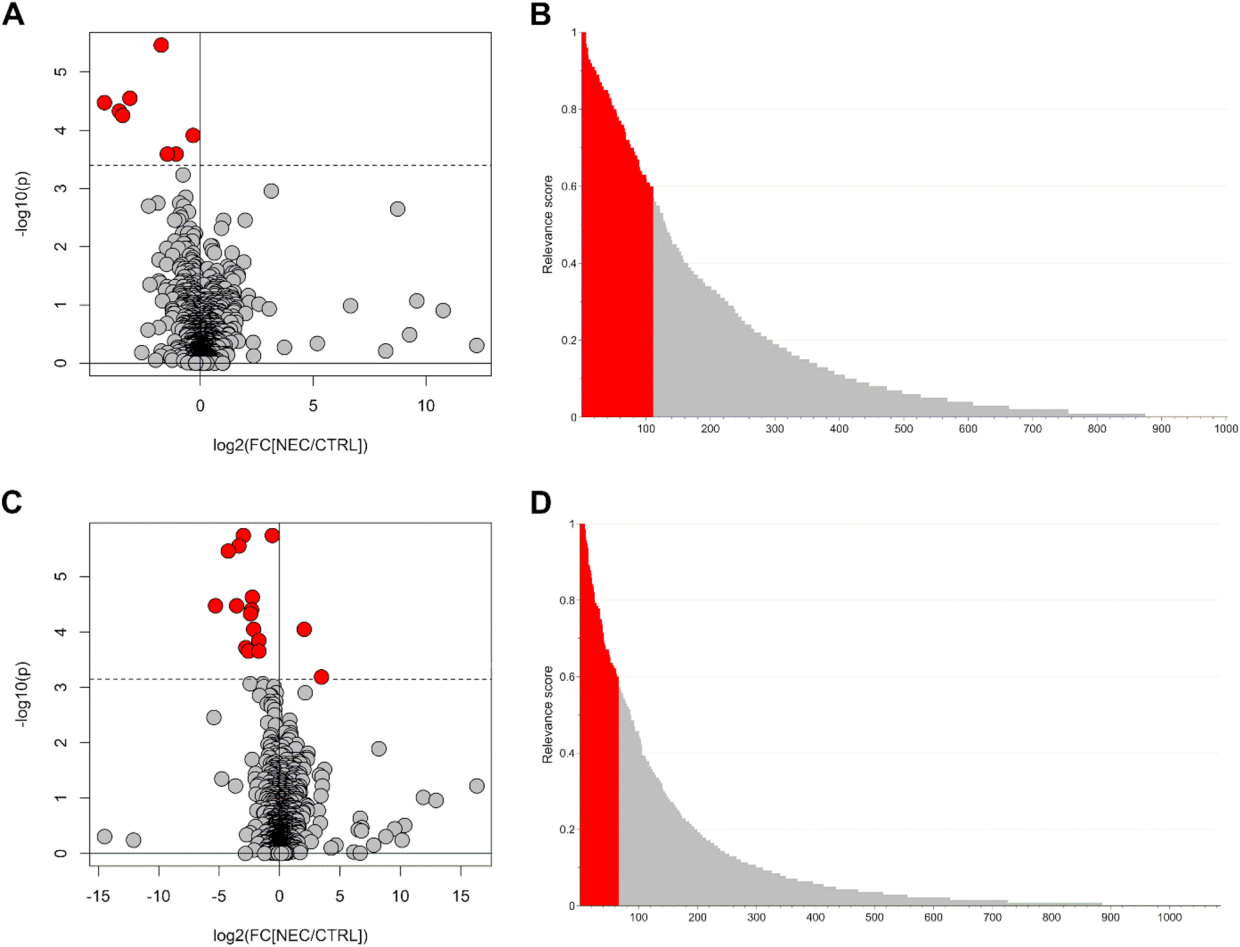
Metabolomics analysis of urine collected at birth (T0): volcano plot (panel **A**) and relevant score plot (panel **B**) obtained for the NEG dataset, and volcano plot (panel **C**) and relevant score plot (panel **D**) obtained for the POS dataset. The features discovered as relevant are colored in red. In the volcano plot, p is the *p*-value of the Mann–Whitney test and FC[NEC/CTRL] is the fold change calculated as ratio between the median in the NEC group and the median in the control group; the dashed black line represents the threshold used to control the false discovery rate

Considering only the comparison of the NEC group and the control group, the Mann‒Whitney test controlling the FDR at the 0.05 level revealed 8 and 16 metabolic variables from the NEG and POS datasets, respectively. The volcano plots in Fig. 2A and C summarize the results. The PLS2C models showed 3 score components for each dataset, with Matthews’s correlation coefficients of cross-validation (MCCcv) of 0.782 (*p* = 0.008) and 0.623 (*p* = 0.020) for the NEG and POS datasets, respectively. The MCCs calculated with the out-of-bag predictions during stability selection (MCCoob) were 0.664 and 0.596, respectively. Assuming a significance level of 0.05, 111 and 66 features from the NEG and POS datasets, respectively, were relevant for distinguishing the two groups. The relevant scores calculated for the datasets are reported in Fig. 2B and D.

Merging the results from the univariate and multivariate data analyses revealed 111 and 67 relevant features, respectively, for the NEG and POS datasets.

#### Metabolome evolution in the first 28 days of life

Urine samples were collected from eighteen subjects in the NEC group and 14 controls at all three time points in the experimental design.

**Fig. 3:**
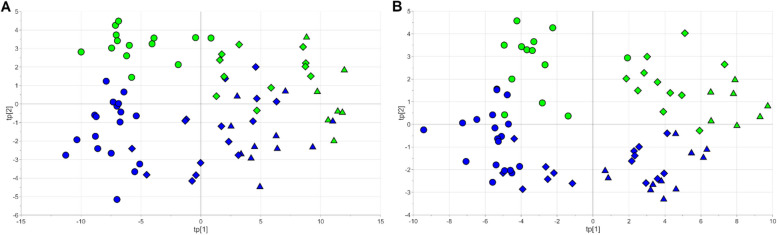
PLS-doe: score scatter plots of the models obtained with the NEG (panel **A**) and the POS (panel **B**) datasets. Samples of the NEC group (blue) and those of the controls (green) clusterise according to the group at all time points along tp[2], while the time increases from left to right along tp[1]. Circles are used for samples at T0, diamonds for samples at T1 and triangles for samples at T2

Exploratory data analysis based on PLS-Does revealed that the urinary metabolome changed over time but that there was a difference between the NEC and control groups. Indeed, a PLS-doe model with 3 score components, R^2^ = 0.71 (*p* < 0.01) and Q^2^ = 0.57 (*p* < 0.01), was obtained for the NEG dataset, and a model with 4 score components, R^2^ = 0.79 (*p* < 0.01) and Q^2^ = 0.63 (*p* < 0.01), was calculated for the POS dataset. The score scatter plots of the two models are reported in Fig. 3.

**Fig. 4:**
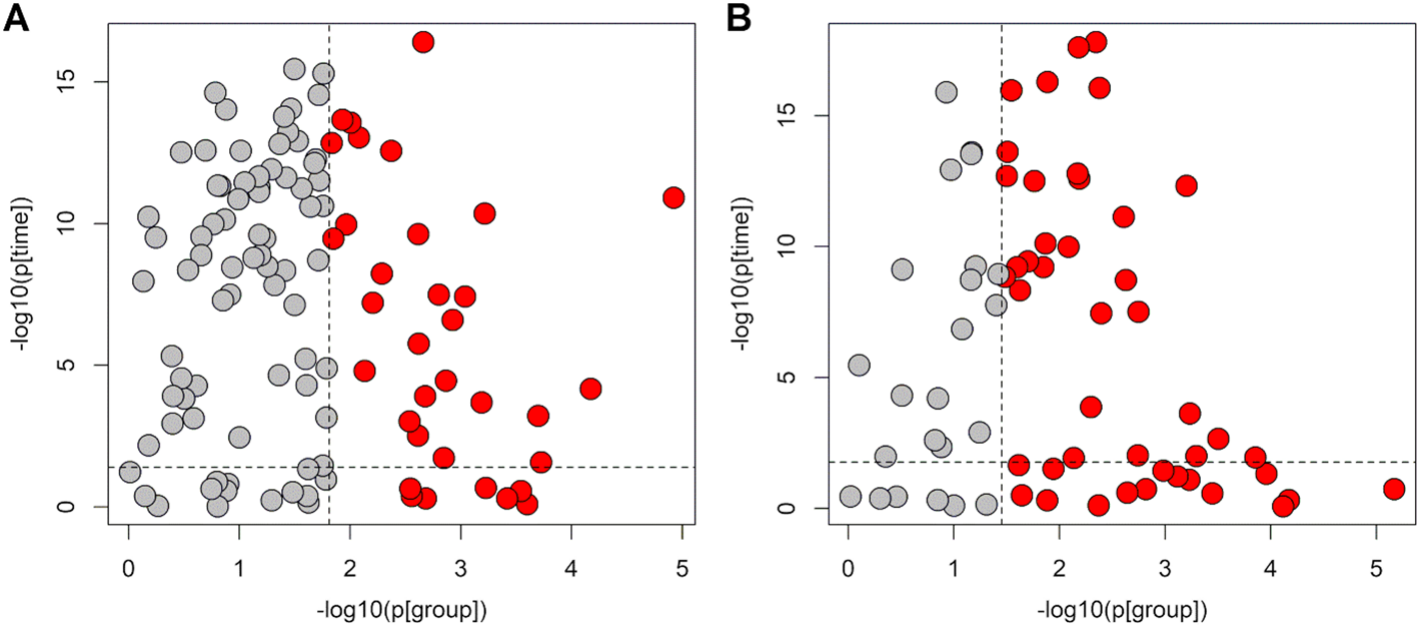
LME models for longitudinal data: NEG dataset (panel **A**) and POS dataset (panel **B**); p[time] and p[group] are the *p*-values of the fixed effects “time” and “group”, respectively. Features significantly relevant to distinguish NEC cases and controls are in red. The dashed black lines indicate the thresholds used to control the FDR at level 0.05

LME analysis controlling the FDR at a level of 0.05 was applied to the set of relevant metabolites discovered at birth. Thirty-three and 44 variables for the NEG and POS datasets, respectively, were found to be significant in distinguishing the two groups throughout the first 28 days of life (Fig. 4).

By searching the Human Metabolome Database (HMDB), variable annotation led to 12 variables being annotated at level 3. Among these, mevalonic acid and N-acetylcystathionine and their isomers had FC[NEC/CTRL] < 0.4, which was reduced in NEC patients compared to controls (Table S2 of the Supplemental material).

#### Prediction of NEC severity from urine at birth (T0)

A comparison between surgical and medical NEC patients was performed considering urine at T0. To avoid bias in the analysis due to differences in the clinical data, 7 patients with medical NEC were matched to 7 patients with surgical NEC (Table S3 of the Supplemental material).

**Fig. 5:**
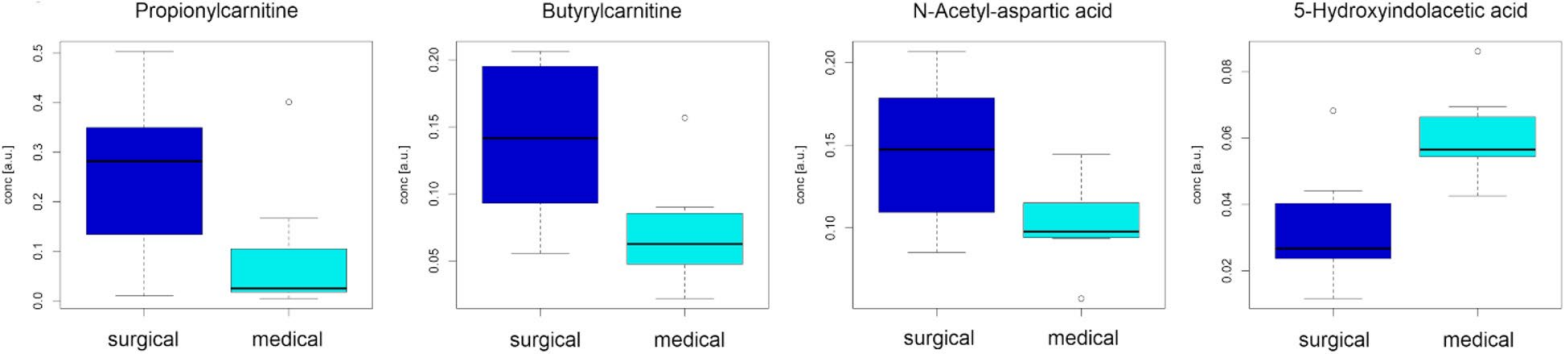
Boxplots representing the distributions of the most significant metabolites discovered in the comparison of medical vs surgical NEC at T0

To limit redundancy in the data and spurious results, the analysis focused on the behavior of 89 annotated metabolites at level 1 of the HMDB, which were identified and used to characterize the two groups. Univariate data analysis revealed 2 metabolites with a *p* value < 0.10. Multivariate analysis based on PLS2C generated a model with 1 score component, with an MCCcv of 0.577 (*p* = 0.036) and an MCCoBoB of 0.500. Combining the two analyses, 6 metabolites were relevant for distinguishing the two groups (Table 3, Fig. 5). In particular, N-acetyl-aspartic acid, butyrylcarnitine and propionylcarnitine were increased in patients with surgical NEC, whereas 5-hydroxyindolacetic acid was decreased in these patients.

**Table 3:** The relevant metabolites in the comparison of medical vs surgical NECs were as follows: annotation is the name of the compound; FC[medical/surgical] is the fold change at T0 calculated as the ratio between the median in the medical NEC group and the median in the surgical NEC group; and p is the *p* value of the Mann‒Whitney test

Annotation	FC[medical/surgical]	*p*
5-Hydroxyindolacetic acid	2.12	0.017
N-Acetyl-aspartic acid	0.66	0.073
Butyrylcarnitine (C4 carnitine)	0.44	0.128
Propionylcarnitine (C3 carnitine)	0.09	0.165
Taurohyocholic acid	0.80	0.209
N-Acetyl-glycine	1.07	0.209

## Discussion

NEC remains one of the most common yet inexplicable causes of death in preterm infants [2]. Its multifactorial pathogenesis makes it challenging to identify a single biomarker of this disease [13, 14, 37, 38]. Rapid, bedside, point-of-care tests, which can be performed prior to or at clinical manifestations, may help guide management, for instance, through feeding strategies, appropriate administration of antibiotics, or the need for urgent surgical intervention.

With their hypothesis-free hypothesis-generating approach, “omic” technologies may untangle a better understanding of the molecular processes responsible for NEC. Several studies using the proteomic approach have already demonstrated to be capable of providing highly accurate diagnostic and prognostic information for infants with suspected NEC [39, 40]. Metabolomics has several advantages over other omic approaches because it detects the functional end points of cellular reactions and the direct results of a biochemical response to a stimulus. As the last downstream products of gene transcription and enzymatic pathways, metabolites provide a closer picture of an organism’s phenotype and its interaction with the environment [16]. Several reviews in the last 10 years have summarized studies using metabolomics for the detection of NEC biomarkers [20, 26, 38, 41], revealing wide variability in population inclusion criteria, the definition of NEC, the timing of sample collection (encompassing early samples or samples close to NEC onset), and the type of analysed biological fluid (plasma, urine, or stools).

As in previous similar studies, our study had a cross-sectional design with a longitudinal collection of data and samples and subsequent sample analysis.

To exclude biases caused by the possible influence of perinatal and postnatal factors on the individual’s metabolome, a strict selection of subjects was applied, and only groups matched for the major clinical characteristics were considered for the metabolomic analysis.

First, we applied one-class classification using MPA to develop prediction models for diseases with respect to a healthy state. The MPA is a powerful tool for modelling because it permits variable selection, outlier detection, and model comparison, thus improving the prediction of the model [32]. By this statistical analysis, we found that metabolomic analysis of early urine samples was able to correctly identify patients who developed NEC, SIP or other GI diseases with respect to controls, with a good prediction rate. Therefore, the urinary metabolome appears to be closely associated with underlying disease beginning very early in life.

Similarly, both univariate and multivariate analyses were able to generate good prediction models distinguishing NEC patients from controls based on relevant metabolic features from early urine samples. Indeed, the Matthews’s correlation coefficients of the models (MCCcv and MCCoob) were > 0.59 (an explanation of these scores is described in the Supplemental Material). Of these significant metabolites that emerged at T0, 77 remained differentially expressed between the two groups throughout the first 28 DOLs. By searching HMDB, 12 of these variables were annotated at level 3 (meaning that further analysis with mass spectral fragmentation should be performed to have a more robust hypothesis of the metabolite).

In particular, mevalonic acid (MVA) was significantly lower in NEC patients than in controls (FC[NEC/CTRL 0.12]). This hydroxy fatty acid, produced by 3-hydroxy-3-methylglutaryl-CoA reductase (HMGCR), is crucial for the biosynthesis of cholesterol, as are sterol and nonsterol isoprenoids (i.e., ubiquinone, vitamin K, isopentenyl t-RNA, heme A, and farnesyl and geranyl lipid anchors). Interestingly, MVA pathway blockade/deregulation has been linked to mitochondrial dysfunction with the consequent release of pro-apoptotic factors, defective autophagy, and possibly inflammasome activation with cell death (which occurs in the case of Mevalonate Kinase Deficiency MKD deficiency). We can hypothesize that similar consequences in the gut of infants affected by NEC due to an impaired MVA pathway result in increased inflammation [42, 43].

A further interesting result of our study is the discrimination of surgical vs medical NEC from urine collected within the first 2 DOLs. In particular, 6 variables were relevant for distinguishing these two groups, with increased N-acetylaspartic acid, butyrylcarnitine and propionylcarnitine in infants with severe disease needing surgery.

N-acetylaspartic acid (NAA) is an amino acid predominantly found in the brain and in lower amounts in peripheral organs. Upregulation of its pathway has been reported in Canavan disease (a childhood leukodystrophy), Parkinson’s disease and type 2 diabetes [44]. Recent studies suggest that the upregulation of NAA leads to oxidative stress with increased nitric oxide and reduced potential antioxidants in rats [45, 46]. Although the role of this metabolite is difficult to interpret in NEC, alterations in acylcarnitine (AC) profiles, such as those of butyrylcarnitine and propionylcarnitine (C4 and C3), have already been described under these conditions. By targeted analysis of blood spots (newborn screening test), Sylvester et al. found 14 acylcarnitine levels to be associated with the risk of NEC, especially within the first 48 h of life [47]. The same group showed that altered levels of certain ACs on day 1 were associated with NEC development [48]. Despite the debated function of ACs in the intestinal epithelium, it has been hypothesized by previous authors that poor GI motility and a vulnerable mucosa (as in the preterm gut), in addition to carbohydrate malabsorption, the presence of lactose from infant formula, and bacterial overgrowth, may cause gut fermentation of excessive carbohydrates to short-chain fatty acids (SCFAs), from which ACs are derived. These factors, in turn, could impair the clearance of intraluminal content and reduce the intraluminal gut pH, favouring a microbial community imbalance, proliferation of bacteria and their translocation through the mucosa with consequent necrosis [47–50]. Thus, the increase in ACs, as a reflection of altered beta-oxidation of FAs, may be a proxy for metabolic prematurity, indicating a greater risk of abnormal responses to metabolic challenges, such as feeding [47, 51]. As in Sylvester et al. [47], in our study, changes in AC concentrations appeared to be chain length specific, with increases in short-chain acylcarnitines (C3 and C4) conferring the risk of severe NEC.

Considering the small number of metabolomic studies on NEC and their great heterogeneity with respect to enrolled patients, diagnostic criteria, collected biofluids and analytical methods, an adequate comparison of our results to those previously published is difficult [20, 25, 37, 38]. Studies applying untargeted metabolomics for NEC have utilized NMR spectroscopy [23–25] or UPLC-MS on plasma [27], urine or stools [21, 22]. The majority of studies collected samples in proximity to the NEC diagnosis, i.e., samples were diagnostic for NEC or could be used to predict NEC severity. Few studies have compared infants with NEC with those with LOS [27, 52, 53] or with feeding intolerance [25]. However, to our knowledge, no studies have evaluated the metabolic differences between patients with NEC and patients with SIP, which is the main disease in differential diagnosis [20]. Only one study assessed the differences in the gut microbiota between infants with NEC and those with SIP on formalin-fixed paraffin-embedded tissue from the site of disease [54].

In urine, Morrow et al. [23] demonstrated that an early (4–9 DOL) alanine/histidine ratio > 4 was associated with microbial characteristics and had a sensitivity of 82%, with a predictive value of 78% for NEC. Additionally, alanine was positively associated with NEC cases that were preceded by Firmicutes dysbiosis, and histidine was inversely associated with NEC cases preceded by Proteobacteria dysbiosis, indicating a close link between the gut microbiota and metabolomic signatures.

Picaud et al. [25], instead, applied NMR spectroscopy to urine collected before, during and after the diagnosis of Bell’s stage II NEC (6 patients) and demonstrated that lactate, betaine, myo-inositol, urea, creatinine, and N,N-dimethylglycine discriminated late-onset NEC (> 3 weeks of life) from controls with good feeding tolerance.

Thoimadou et al. [24] applied both untargeted NMR spectroscopy and targeted LC‒MS to urine samples collected after initial evaluation for NEC (15 cases, every stage) and at a similar postnatal age for controls. They found 25 discriminant metabolites, with NEC patients characterized by lower levels of tyrosine, proline, citrate, 4-hydroxybenzoate, formate, succinate, 4-hydroxyphenylacetate, fumarate, creatinine, myoinositol, and hippuric acid.

In 2022, the same authors [27] found significant differences in the metabolic profile of neonates with LOS or NEC compared to controls by analysing their serum with LC-quadrupole time-of-flight MS. Phosphatidylcholines or lysophosphatidylcholines were significantly reduced both in neonates with LOS and those with NEC compared to controls, whereas L-carnitine could efficiently discriminate NEC patients from controls.

Methodological inconsistencies among studies, disagreement on conventions for data reporting and analysis, and the sheer magnitude of data produced are significant drawbacks to studies applying high-throughput multiomics technologies. To overcome these challenges, machine learning and artificial intelligence are becoming popular for integrating omics data with NEC clinical features, progression phenotypes, and predicted therapeutic targets, resulting in clinically meaningful information.

The major strength of our study is that we applied a strict selection of subjects to eliminate biases in the metabolomic analysis. Additionally, we included infants affected by SIP or other GI diseases in the differential diagnosis of NEC. Third, we focused on the prediction of NEC severity and identified potential biomarkers of surgical NEC. Finally, we collected samples at multiple timepoints throughout the first month of life, starting at birth. This has permitted the evaluation of the evolution of the metabolome through time, given its potential changes related to enteral and parenteral feeding, infections, administered drugs and comorbidities.

Regarding limitations, the small number of patients has hampered the analysis of patients with only definite NEC (Bell’s stage ≥ II). Also, we could not perform the analysis of samples collected at the time of NEC diagnosis. Despite this, to our knowledge, our cohort is one of the largest enrolled in an untargeted metabolomic study on NEC thus far [20]. Our future directions will be to develop integrated models that combine metabolomic biomarkers with clinical parameters, as well as to validate the identified features and model performance with independent datasets to confirm the generalizability of our results.

## Conclusion

This explorative study demonstrated that the urinary metabolome is closely associated with underlying gastrointestinal disease beginning very early in life. Furthermore, urinary metabolic features characterize NEC patients vs healthy controls from birth until 28 days of life. Finally, untargeted metabolomics can identify patients who will develop surgical NEC as early as birth, with a potential role for impaired fatty acid and acylcarnitine metabolism.

Our study confirms the important role of metabolomics in the investigation of disease pathophysiology and in the discovery of potential diagnostic/prognostic biomarkers of NEC. Our results need to be validated in future studies on independent cohorts and using targeted analysis.

## Supplementary Information

Supplementary Material 1.

## Data Availability

Clinical and metabolomic data of this study have been deposited in Mendeley Data Repository (Moschino, L; Stocchero, M (2024), “METNEC Study”, Mendeley Data, V1, 10.17632/83myp4xfcn.1).

## Abbreviations

ATB: Antibiotics
BW: Birth weight
CRP: C-reactive protein
DOL: Day of life
ELGANs: Extremely low gestational age neonates
EOS: Early onset sepsis
ExLap: Exploratory laparotomy
FEF: Full enteral feeding
GA: Gestational age
GI: Gastrointestinal
HsPDA: Haemodynamically significant patent ductus arteriosus
IUGR: Intrauterine growth restriction
MS: Mass spectrometry
NEC: Necrotizing enterocolitis
NMR: Nuclear magnetic resonance
NPO: Niil per os
PD: Peritoneal drainage
PROM: Premature rupture of membrane
SIP: Spontaneous intestinal perforation
UPLC: Ultra-performance liquid chromatography
WBC: White blood cells
VLBW: Very low birth weight infants
