# Supplementary material for: Metabolomic analysis to predict the onset and severity of necrotizing enterocolitis
Source: BMC Gastroenterol. 2024 Oct 26;24:380. doi: 10.1186/s12876-024-03453-y (PMC11515140; doi:10.1186/s12876-024-03453-y)
Supplement: Supplementary file 1 — Supplementary Material 1. [file 12876_2024_3453_MOESM1_ESM.docx]

METABOLOMIC ANALYSIS TO PREDICT THE ONSET AND SEVERITY OF NECROTIZING ENTEROCOLITIS

Laura Moschino^1,2^, Giovanna Verlato^1^, Matteo Stocchero^2,3^, Giuseppe Giordano^2,3^, Paola Pirillo^2,3^, Marta Meneghelli^1^, Silvia Guiducci^1^, Miriam Duci^4^, Francesco Fascetti Leon^4^, Eugenio Baraldi^1,2^

^1^Neonatal Intensive Care Unit, Padova University Hospital, Italy

^2^Institute of Pediatric Research, Città della Speranza, Italy

^3^Laboratory of Mass Spectrometry and Metabolomics, Institute of Pediatric Research, Padova University Hospital, Italy

^4^Pediatric Surgery, Padova University Hospital, Italy

# **MATERIALS AND METHODS**

## Collection of clinical data and samples

For each patient clinical and demographic characteristics, as well as laboratory data, were recorded on a preformed electronic case report form (eCRF) on the REDCap platform. An identification code was assigned to each subject, in order to protect confidentiality according to the rules of good clinical practice and the Dlgs 196/2003. Plasma, urine and faecal samples were non-invasively collected at birth (within 48 hours, T0), at 14 (T1) and 28 DOL (T2), at 2 months (T2 months) and at 36 weeks of corrected gestational age (cGA) (T36). In the suspicion of NEC, SIP or other GI diseases with a presentation similar to NEC, additional plasma, urine and faecal samples were collected at symptoms’ onset and then weekly until resolution. Plasma samples were obtained either from blood samples drawn through arterial or venous lines already in place, or from spare capillaries drawn for routine haematocrit and bilirubin levels. Urine was collected using a cotton wad and then transferred into a tube. Faeces were collected from the infants’ nappies using a clean pad and then transferred into a tube.

## Untargeted metabolomic analysis

Sample preparation

Urine samples were initially stored in a freezer at -80°C until metabolomic analysis. Then, they were slowly thawed overnight at +4°C and then transferred to ambient temperature for the preparation. Each sample was stirred and centrifuged at 3000 rpm (1509 g) for 15 min at 10°C, then 20 μl of the supernatant from each sample were pipetted in a well of 384 wells plate, adding 280 μL of 0.1% formic acid (FA) solution (finale volume 300 μL, dilution 1:15). All the procedures for the preparation were automatically managed by a robotic liquid handling system, Multiprobe II Ex (Perkin Elmer).

Experimental setting

Untargeted metabolic profiling of urine samples was performed in positive and negative electrospray ionization (ESI+, ESI-) mode on an Acquity Ultra Performance Liquid Chromatography (UPLC) system (Waters, U.K.) coupled to a Quadrupole Time-of-Flight (QToF) Synapt XS HDMS mass spectrometer (Waters MS Technologies, Ltd., Manchester, U.K.).

The mass range scan was of 20 to 1200 amu, in MS scan mode. The capillary voltage was set at 0.7 KV; and the sampling cone voltage at 40 V. The desolvation gas flow was set at 800 L/h with temperature kept at 400°C. The cone gas flow was set at 20 L/h with temperature kept at 110°C. To correct for changes in environmental or experimental condition over the course of the analysis, Leucine-Enkephalin solution at a concentration of 100 pg/ml was injected periodically (every 30 s) as internal reference (i.e. lock mass).

For LC-MS analysis a Waters Acquity UPLC HSS T3 column 2.1 mm wide and 100 mm long packed with 1.8 μm beads was used and its temperature was kept at 50°C. The mobile phase flow rate was set at 0.5 ml/min. The gradient mobile phase consisted of water with 0.1% FA (A) and methanol with acetonitrile in a 90:10 ratio with 0.1% FA (B). Each sample run lasted 12 minutes and consisted of an isocratic phase of 5% B for 1 minute, a linear increase to 30% B in 2.5 minutes, a linear increase to 95% B in 3 minutes, an isocratic phase of 95% B for 1.5 minutes, a washout phase of 5% B for 3 minutes. For each run, 3 μl of sample were injected.

Quality Control samples (QC), blanks and Standards Solution Samples (Mix) were used to assess reproducibility and accuracy during the analysis, and examine the metabolite content of the samples. The QCs were prepared from an aliquot (10 μL) of each sample, pooled together and diluted with eight different dilution factors (1:3, 5, 7, 10, 15, 20, 50, 100) with 0.1% FA solution in water, treated as the samples. The Mix consisted of nine compounds of known exact mass and retention time. The QCs and Mixes were injected at regular intervals of 15 samples during the sequence, together with blank samples, to identify specific ions from the mobile phase, and any contaminants.

The sequence for the analysis was randomized to prevent any spurious classification deriving from the position of the sample in the sequence.

Data pre-processing

Raw data were extracted using Progenesis QI software (Waters Corporation, U.S.A.). The parameters were optimized through the preliminary processing of the QCs. Specifically, 0.5 was set as filter to import the raw data in positive ionization (ESI+) and 0.3 for negative ionization (ESI-), and the QC in the middle of the sequence was selected as reference for the automatic retention time alignment of the samples in the sequence. The sensitivity of the automatic algorithm for peak picking was set at 3 for both ionizations, in the time range from 0.4 to 8.0 min. As a result, the so-called time_mass variables (where “time” is the retention time and “mass” is the mass to charge ratio m/z of the spectral feature) were generated.

Features with at least one missing data in the QCs and more than 10% of missing data in the samples

were eliminated. For each variable passing such a filter, missing data were imputed with a random

number between zero and the minimum value measured for that variable. Data were calibrated on

the basis of the local linear regression models obtained considering the trend of the QCs with the run order. Probabilistic quotient normalization was applied to take into account dilution effects.

Variables with a coefficient of variation greater than 25% in the QCs were excluded.

Data were log-transformed and autoscaled prior to performing data analysis.

## Statistical analysis

Categorical clinical data were investigated by Fisher’s exact test, whereas continuous clinical data by t-test for data with a normal distribution or Mann-Whitney test in the case of data with a non-normal distribution. Normal distribution was assessed by Shapiro-Wilk test, assuming a significant level of 0.10.

Since the urinary metabolome is strongly dependent on the perinatal and neonatal characteristics

of the subjects, a suitable procedure of matching was applied to avoid bias in the metabolomics

analysis. The procedure is based on pairwise distances and reduce the confounding among the

factors included in the experimental design (1).

The urinary metabolome was investigated using different approaches.

One-class classification (OCC) was applied to model the controls in order to build a classifier able to assess if a new sample belongs or not to the control group. Specifically, two different methods, one based on K-Nearest Neighbors (KNN) and one on Bounding Box (BB), were included in a procedure that implements Model Population Analysis (MPA) (2). The idea underlying MPA is that information about a data set is not discovered by a single model, but through the analysis of a large population of sub-models generated from a random sampling of the data, permitting variable selection, outlier detection, and model comparison (2,3) and thus improving the prediction and interpretation of the model. (2,3). In our study, the control group was sampled a large number of times by Binary Matrix Sampling (BMS), sampling both observations and features, to extract a large number of subsets that were used to build a large number of KNN and BB-based one-class classifiers.

Thus, the belonging to the control group of the out-of-bag samples was predicted applying the set of one- class classifiers. For each type of classifier, the frequencies of right prediction were considered to estimate the thresholds to use to assess if a new sample belongs or does not belong to the control group. The main advantage of using MPA with respect to the use of a single one-class classifier built on the whole dataset is that it avoids models of the reference data that describe a too large space with the risk of increasing the rate of false belonging to the reference group. The classifier based on KNN considers the distance matrix of the new observations with respect to the control group, and compares the mean distances of the K-nearest controls with a suitable threshold calculated by MPA. The distance matrix is calculated using the scores of the Principal Component Analysis (PCA) model (4) of the data of the control group. Moreover, the Q-distance of the PCA model is used as second score for comparison, and it is compared with the maximum value calculated for the reference data. Values of mean distance and Q-distance exceeding the related thresholds indicate observations that do not belong to the control group. In the BB control chart, the data distribution of each feature in the control group is modelled by Gaussian kernel density estimation and used to calculate the p-value of the data of the new observations. False Discovery Rate (FDR) is applied to detect the number of features for which the new data are outliers for a given significant level. If at least one feature is detected, the observation is considered not belonging to the control group.

The results obtained applying the two one- class classifiers are combined in a consensus approach assuming that an observation does not belong to the controls if at least one classifiers discovers the observation as outlier. In MPA, 500 subsets were generated with a probability of 0.8 and 0.5 for the observations and the variables, respectively, FDR was controlled at level 0.05 in BB and a significance level of 0.05 was assumed in the threshold estimation. Two class comparison was performed as explained in the following. Both univariate data analysis controlling the false discovery rate by Benjamini-Hochberg procedure(5) and multivariate data analysis based on PLS for classification (PLS2C) (6) were applied. In PLSC data modelling, the number of score components of the model was determined on the basis of the first maximum of the Matthews’s correlation coefficient (MCC) calculated by repeated 10-fold full cross-validation (MCCcv) under the condition to pass the randomization test on the class response. Moreover, stability selection based on Variable Influence on Projection (VIP) was used to discover the set of relevant features(7).

Specifically, 200 subsets were randomly extracted considering a probability of 0.7 and 0.5 for the observations and the variables, respectively, for the sampling procedure based on BMS. Additionally, MCCoob was calculated as well. Out-of-bag (OOB) evaluation is a method of measuring the prediction error or the prediction score of random forests, boosted decision trees, and other machine learning models utilizing bootstrap aggregating (bagging). It is used to assess the quality of a model. Compared to cross validation, OOB evaluation uses a sample of remaining data that was not necessarily used during the model's analysis, thus considering a more random sample than the validation set of cross validation. Therefore, MCCoob score represents more accurately a good or worse prediction of the given model (8,9).

PLS for designed experiments (PLS-doe)(10,11) and Linear Mixed-Effects modelling (LME) for longitudinal data (12) controlling the false discovery rate by Benjamini-Hochberg procedure were applied to study the time evolution of the urinary metabolome at T0, after 14 days (T1) and after 28 days (T2). In LME modelling, the fixed effects were “time” and “group” of the subject. A random effect was considered both for the coefficient of time and for the subject. In PLS analysis, the effect of the subject was removed from the data using the random effect estimated by LME and the remaining data matrix was submitted to PLS-doe using as responses the fixed factors “time” and “group” (11)

Data analysis was performed by R-functions developed in-house using the platform R 4.2.2 (R Foundation for Statistical Computing).

# **RESULTS**

**Table S1.** Clinical data of the selected NEC group and Control Group, matched for the reported perinatal and neonatal characteristics to perform the metabolomic analysis at T0. Data with a normal distribution are reported as mean (SD), data with a non-normal distribution as median [IQR] and categorical data as count.

| **Clinical data** | **NEC (N=20)** | **Controls (N=17)** | **p** |
| --- | --- | --- | --- |
| Gestational age | 187.2 (18) | 194.9 (14) | 0.16 |
| Body weight | 793 [289] | 950 [250] | 0.12 |
| Sex (M/F) | 15/5 | 9/6 | 0.49 |
| Delivery mode (vaginal/caesarean) | 2/18 | 0/17 | 0.49 |
| Apgar 5 min | 7 [1.5] | 8 [2.3] | 0.23 |
| PPROM | 3 | 4 | 0.68 |
| IUGR | 5 | 2 | 0.42 |
| EOS | 7 | 4 | 0.56 |
| HSPDA | 11 | 12 | 0.33 |
| AED | 5 | 1 | 0.19 |
| Prenatal steroids (no/incomplete/complete) | 2/5/13 | 2/2/13 | 0.59 |
| Surfactant | 16 | 14 | 1.00 |
| Outborn/inborn | 1/19 | 2/15 | 0.58 |


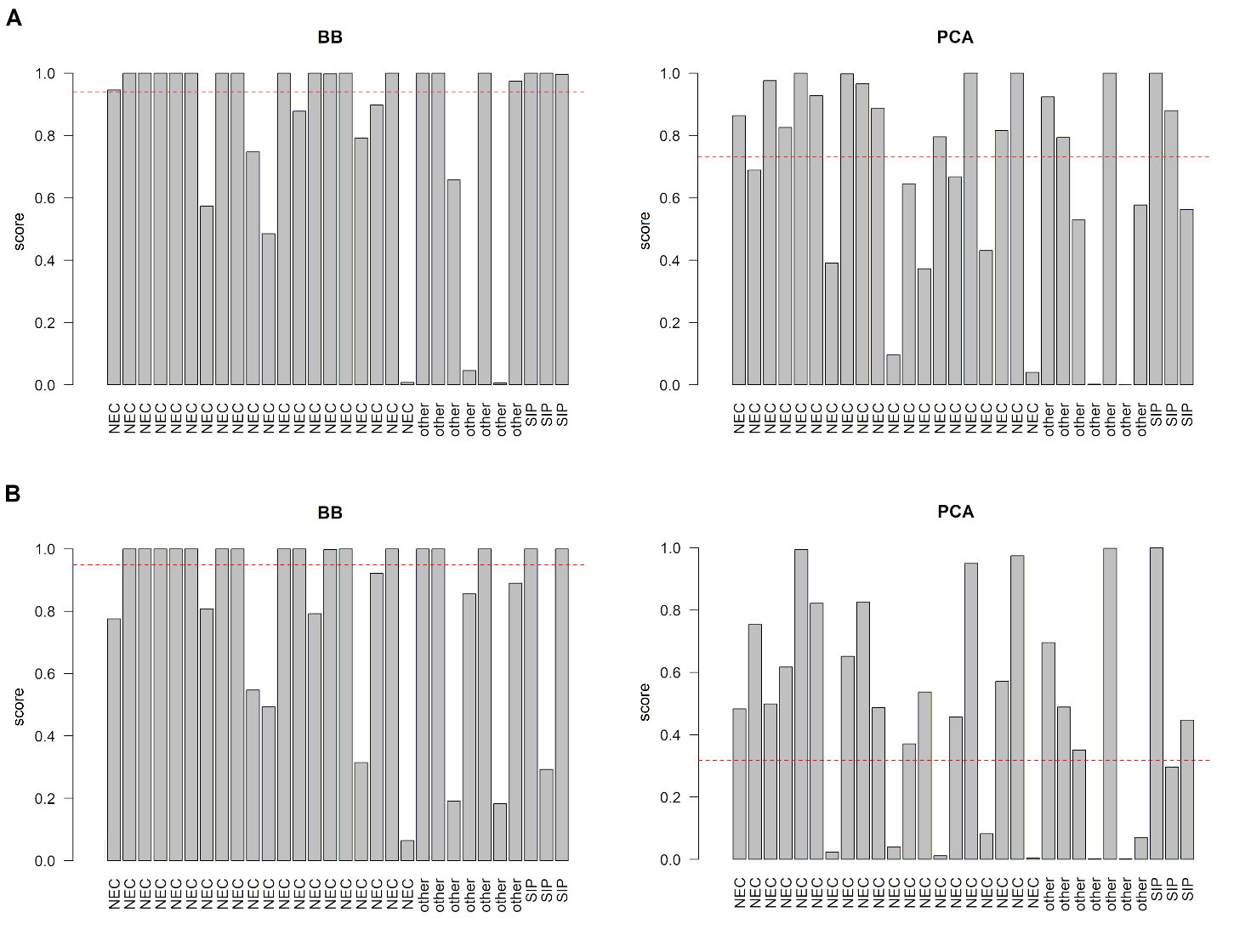


**Figure S1.** Prediction models derived from the control charts using the control group as reference. Panel A shows the classifiers from the NEG dataset, whereas Panel B from the POS dataset. Samples are reported as labels. Samples with score greater than the threshold (dashed red line) in at least one model are correctly predicted as not belonging to the control group.

**Table S2.** Annotated metabolic variables emerged by the comparison between the NEC Group and the Control Group throughout time (first 28 days of life); these variables were annotated at level 3 in the HMDB. ID is the time_mass identifier of the variable, ionization mode specifies the type of ionization, HMDB ID is the identifier of the compound in the Human Metabolome Database, annotation is the name of the compound, HMDB class is the chemical class of the compound and FC[NEC/CTRL] is the fold change at T0 calculated as ratio between the median in the NEC group and the median in the control group.

| **ID** | **ionization mode** | **HMDB ID** | **Annotation** | **HMDB class** | **FC[NEC/CTRL]** |
| --- | --- | --- | --- | --- | --- |
| 2.81_319.1369m/z | ESI + | HMDB00227 | Mevalonic acid | hydroxy fatty acids | 0.125 |
| 0.84_264.0798n | ESI + | HMDB02381 | N-Acetylcystathionine | n-acyl-alpha amino acids | 0.227 |
| 2.63_319.1369m/z | ESI + | HMDB00227 | Mevalonic acid isomers | hydroxy fatty acids | 0.306 |
| 0.97_264.0797n | ESI + | HMDB02381 | N-Acetylcystathionine isomers | n-acyl-alpha amino acids | 0.381 |
| 0.62_470.1510m/z | ESI - | HMDB02061 | Hyaluronic acid | glycosaminoglycan | 0.474 |
| 4.68_364.2258n | ESI + | HMDB06760 | 11beta,17alpha,21-Trihydroxypreg-nenolone | C21-Steroid hormones | 0.491 |
| 0.61_363.0722m/z | ESI + | HMDB02655 | Isorhamnetin | flavonoids metabolites | 0.501 |
| 2.84_267.0958m/z | ESI - | HMDB01563 | 1-Methylguanosine | purine nucleosides | 0.691 |
| 2.84_228.1103n | ESI - | HMDB06695 | Prolylhydroxyproline | dipeptide | 0.767 |
| 1.00_189.0399m/z | ESI - | HMDB00393 | 3-Hexenedioic acid | dicarboxylic acids | 0.813 |
| 1.06_258.0858n | ESI + | HMDB04813 | 3-Methyluridine | modified nucleoside | 0.829 |
| 0.63_175.0243m/z | ESI - | HMDB02545 | Galacturonic acid | organic acids | 0.854 |

**Table S3.** Clinical data of the subjects with medical and surgical NEC who were matched to perform the metabolomic analysis. Data with a normal distribution are reported as mean (SD), data with a non-normal distribution as median [IQR] and categorical data as count.

| **Clinical data** | **Surgical NEC (N=7)** | **Medical NEC (N=7)** | **p** |
| --- | --- | --- | --- |
| Gestational age | 185 [21] | 187 [21] | 0.85 |
| Body weight | 675 [355] | 1000 [380] | 0.22 |
| Sex (M/F) | 5/2 | 7/0 | 0.46 |
| Delivery mode (vaginal/caesarean) | 0/7 | 1/6 | 1.00 |
| Apgar 5 min | 7.0 [1.5] | 7.0 [1.5] | 0.70 |
| PPROM | 1 | 1 | 1.00 |
| IUGR | 2 | 1 | 1.00 |
| EOS | 1 | 2 | 1.00 |
| HSPDA | 4 | 5 | 1.00 |
| AED | 1 | 1 | 1.00 |
| Prenatal steroids (no/incomplete/complete) | 1/2/4 | 0/2/5 | 0.57 |
| Surfactant | 6 | 5 | 1.00 |
| Outborn/inborn | 0/7 | 0/7 | 1.00 |

**REFERENCES**

1. Pierobon ES, Capovilla G, Moletta L, De Pasqual AL, Fornasier C, Salvador R, et al. Multimodal treatment of radiation-induced esophageal cancer: Results of a case-matched comparative study from a single center. International Journal of Surgery. 2022 Mar;99:106268.

2. Deng BC, Yun YH, Liang YZ. Model population analysis in chemometrics. Chemometrics and Intelligent Laboratory Systems. 2015 Dec;149:166–76.

3. Li HD, Liang YZ, Cao DS, Xu QS. Model-population analysis and its applications in chemical and biological modeling. TrAC Trends in Analytical Chemistry. 2012 Sep;38:154–62.

4. Jolliffe IT. Principal Component Analysis. New York: Springer-Verlag; 2002.

5. Benjamini Y. Discovering the False Discovery Rate. J R Stat Soc Series B Stat Methodol. 2010 Sep 1;72(4):405–16.

6. Stocchero M, De Nardi M, Scarpa B. PLS for classification. Chemometrics and Intelligent Laboratory Systems. 2021 Sep;216:104374.

7. Stocchero M. Relevant and irrelevant predictors in PLS2. J Chemom. 2020 Aug 7;34(8).

8. Chicco D, Jurman G. The advantages of the Matthews correlation coefficient (MCC) over F1 score and accuracy in binary classification evaluation. BMC Genomics. 2020 Dec 2;21(1):6.

9. Chicco D, Tötsch N, Jurman G. The Matthews correlation coefficient (MCC) is more reliable than balanced accuracy, bookmaker informedness, and markedness in two-class confusion matrix evaluation. BioData Min. 2021 Feb 4;14(1):13.

10. Stocchero M. PLS for designed experiments. Chemometrics and Intelligent Laboratory Systems. 2023 Sep;240:104928.

11. Peila C, Sottemano S, Cesare Marincola F, Stocchero M, Pusceddu NG, Dessì A, et al. NMR Metabonomic Profile of Preterm Human Milk in the First Month of Lactation: From Extreme to Moderate Prematurity. Foods. 2022 Jan 26;11(3):345.

12. Laird NM, Ware JH. Random-Effects Models for Longitudinal Data. Biometrics. 1982 Dec;38(4):963.
